# Simultaneous Median and Ulnar Compression Neuropathy Secondary to a Giant Palmar Lipoma: A Case Report and Review of the Literature

**DOI:** 10.7759/cureus.2198

**Published:** 2018-02-16

**Authors:** Melih Unal, Engin Demirayak, Baver Acar, Ozkan Kose

**Affiliations:** 1 Department of Orthopaedics and Traumatology, University of Health Sciences, Medical Faculty, Antalya Education and Research Hospital, Antalya, Turkey

**Keywords:** lipoma, median nerve, ulnar nerve, carpal tunnel syndrome, guyon’s canal

## Abstract

Lipomas are benign tumors that rarely settle in the hand. They usually present with mass, pain, and nerve compression symptoms. Although isolated median or ulnar nerve compression neuropathy secondary to a lipoma of the hand has been widely reported, simultaneous median and ulnar nerve compression neuropathy are exceedingly rare and there are only three reported cases in the current literature to date. Herein, a case of a 50-year-old woman with a giant palmar lipoma that caused median and ulnar compression neuropathy is presented. The removal of the tumor resulted in the complete recovery of the patient’s symptoms. A deep-seated palmar lipoma should be kept in mind in patients with unilateral compression neuropathy symptoms with a palmar mass.

## Introduction

Lipomas are benign soft tissue tumors derived from mature adipose tissue. Theoretically, these tumors may involve every organ throughout the entire body where fat tissue is present. However, the hand is not frequently involved. In a retrospective study of the epidemiology of hand tumors, only 20 (4.9%) out of 402 hand tumors were diagnosed as lipomas [1]. A lipoma of the hand can be located in subcutaneous, subaponeurotic, or intramuscular locations. However, a palmar subaponeuratic lipoma is the most common type of location. In a study that reviewed 18 cases of hand lipoma, 14 (78%) cases were located in the deep palmar area [2]. A deep-seated palmar lipoma may cause nerve compression syndromes when settled in the hand due to the limited space. Even a small-sized lipoma may become symptomatic [3]. 

Previously, several nerve compression neuropathy cases of the hand secondary to lipomas have been described and published. Most of these cases involved either the ulnar nerve or the median nerve [3-5]. On the other hand, simultaneous median and ulnar nerve compression neuropathy due to lipomas is exceedingly rare and there are only three reported cases in the current literature to date (Table 1) [6-8]. 

**Table 1:** Previously reported cases with a palmar lipoma causing both median and ulnar nerve entrapment Previously reported cases with palmar lipomas causing both median and ulnar nerve entrapment in the current literature. (Abbreviations, F: Female, M: Male, R: Right, L: Left, Mo: Motor, S: Sensorial)

Case #	Author	Year	Age	Sex	Side	Clinical findings	EMG	Deficit							Duration of symptoms	Treatment	Mass Size (cm)	Outcome	Recurrence	Follow-up
								Ulnar				Median								
								Mo		S		Mo		S						
1	Galeano et al. [6]	2001	49	F	R	Painful mass in her right palm	Increase in the distal sensory latency of the ulnar and median nerves	-	+		-		+		6 Years	Excision + Carpal Tunnel Release	5 x 2	Complete	No	NR
2	Pagonis et al. [7]	2011	63	F	L	Median and ulnar nerve compression symptoms and a prominent mass on her left palm and thenar eminence	Alteration of the normal values due to pressure on both the median and ulnar nerves	+	+		+		+		3 Years	Marginal Excision	8 x 4 x 3.75	Complete	No	30 Months
3	Kamath et al. [8]	2016	49	M	L	Severe tingling sensation involving all the five digits	Increased latency for both motor and sensory modalities for median and ulnar nerves	-	+		-		+		4 Months	Marginal Excision + Carpal Tunnel and Guyon Canal Release	1.5 x 1	Complete	No	6 Months
4	Current Case	2017	50	F	L	Growing mass in the palm and numbness in fingers	Both median and ulnar nerve entrapment in wrist	-	+		-		+		3 Years	Marginal Excision + Carpal Tunnel and Guyon Canal Release	6 x 5 x 4	Complete	No	9 Months

Herein, a further case with simultaneous median and ulnar compression neuropathy secondary to a giant palmar lipoma is presented and discussed.

## Case presentation

**Figure 1:**
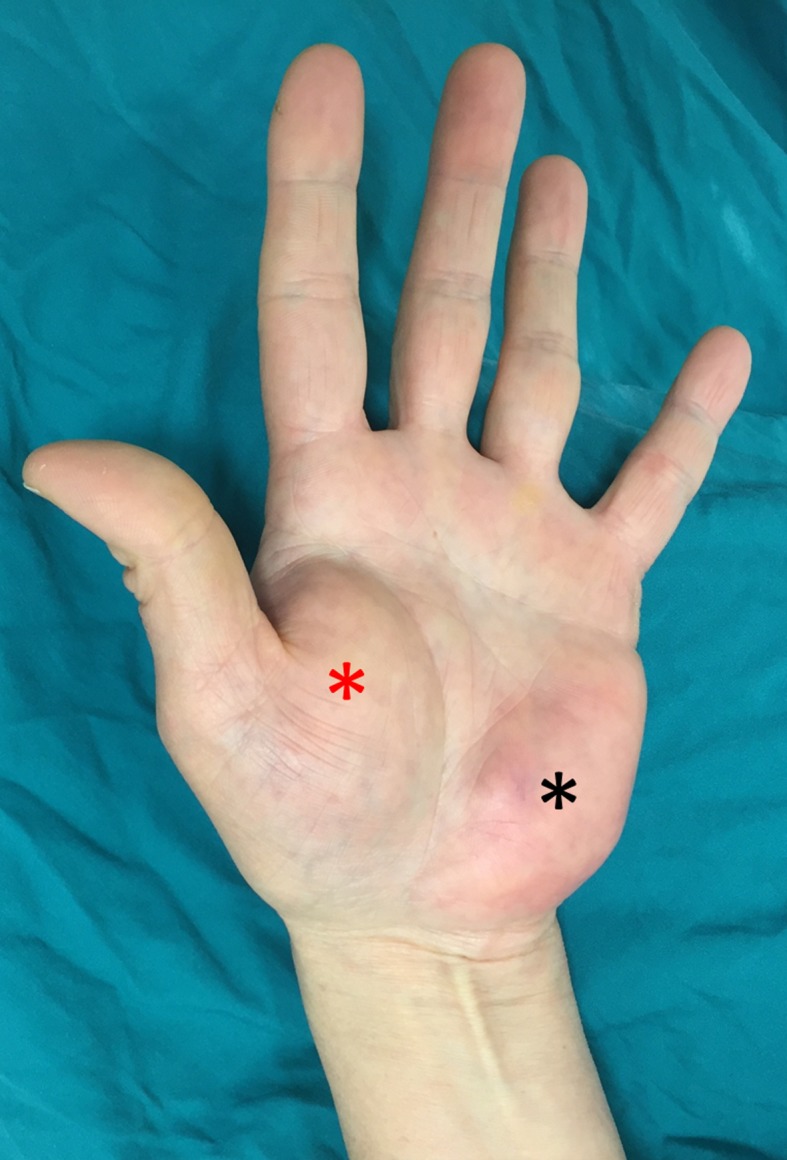
The clinical appearance of the patient’s hand The clinical appearance of the patient’s hand. Note the mass in both the thenar (red asterisk) and hypothenar (black asterisk) regions of the palm.

A 50-year-old female patient presented to our outpatient clinic complaining of numbness in all her fingers and an accompanying mass in her palm. Her complaints lasted for three years but the mass gradually increased in size and numbness deteriorated over the last six months. The patient was otherwise healthy, without any other chronic disease. On physical examination, a palpable rubbery mass both in the thenar and the hypothenar regions of the palm was present (Figure 1).

**Figure 2:**
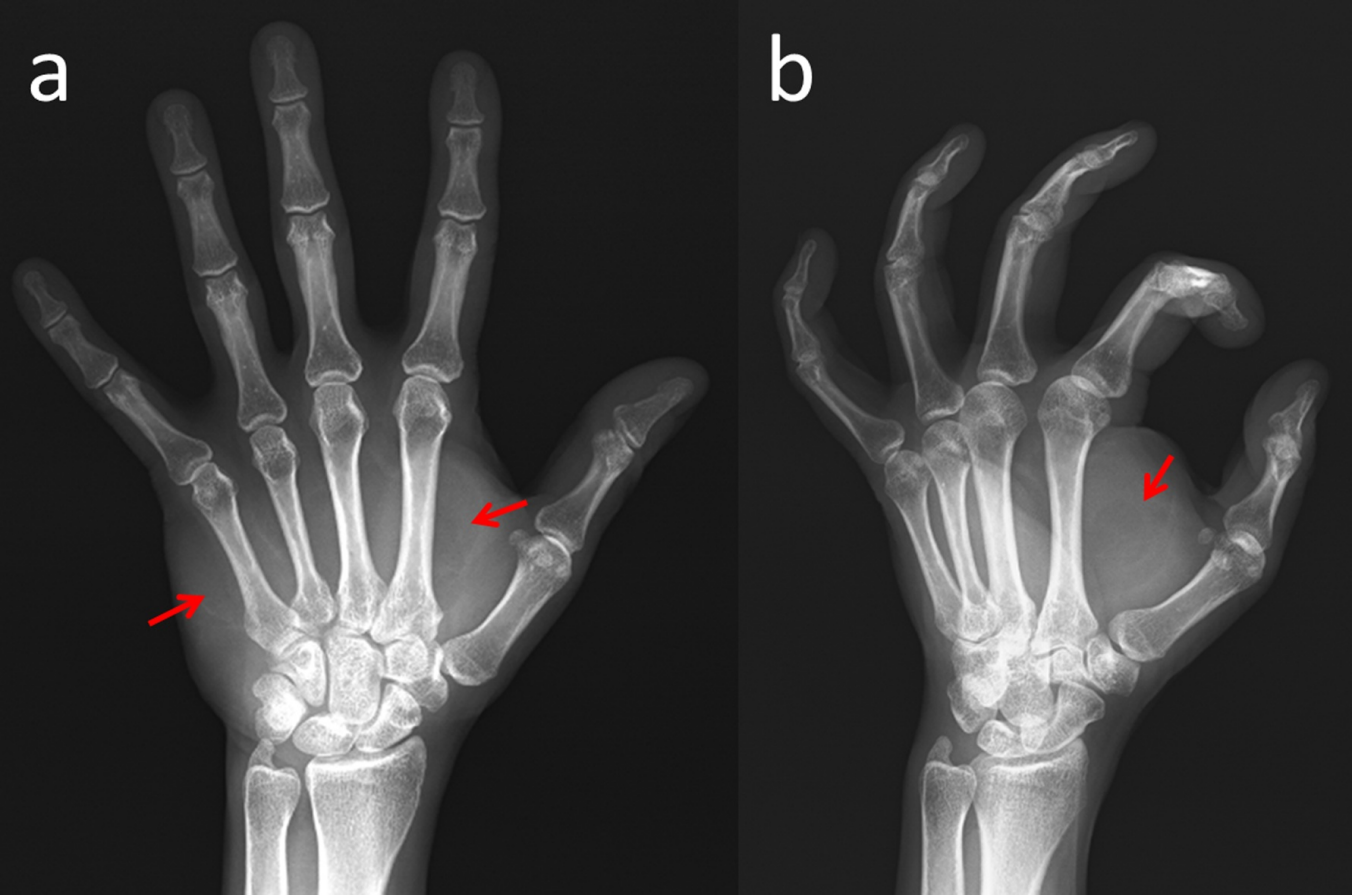
Radiographs of the hand (a) Antero-posterior and (b) oblique hand radiographs show soft tissue shadows in the palmar region (red arrows).

Her range of motion of wrist and phalanges was normal. Radial and ulnar pulses were palpable and capillary refill was normal. Median and ulnar nerve motor examination showed normal findings but sensory examination showed hypoesthesia. Phalen’s test and Tinel's sign on both the carpal tunnel and Guyon’s canal were positive. The hand radiograph revealed no osseous pathology except that the mass covered her palm (Figure 2).

**Figure 3:**
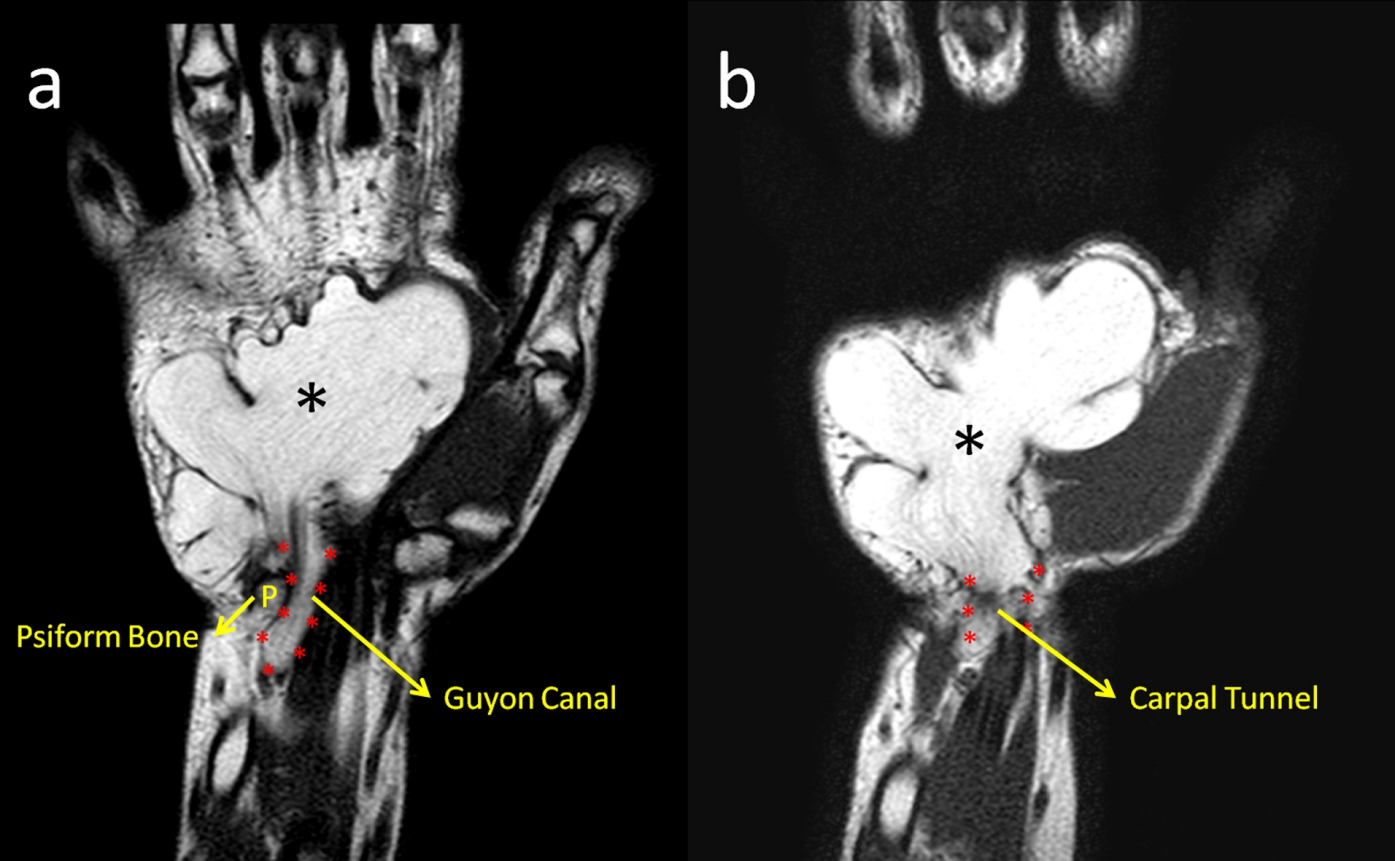
MRI of the hand (a) and (b) T1 weighted coronal MRI sections demonstrated the compression of both Guyon's canal and the carpal tunnel by the giant lipoma (black asterisk). MRI: Magnetic resonance imaging

Magnetic resonance (MR) imaging revealed a giant mass that had similar intensity with subcutaneous fat tissue extending to the palm and showing invasion of the Guyon’s canal and the carpal tunnel (Figure 3).

Electromyography showed both median and ulnar nerve entrapment in the wrist. Based on physical examination, neurophysiological examination, and imaging findings, the lipoma causing both median and ulnar nerve compression neuropathy was diagnosed and surgical excision was planned.

**Figure 4:**
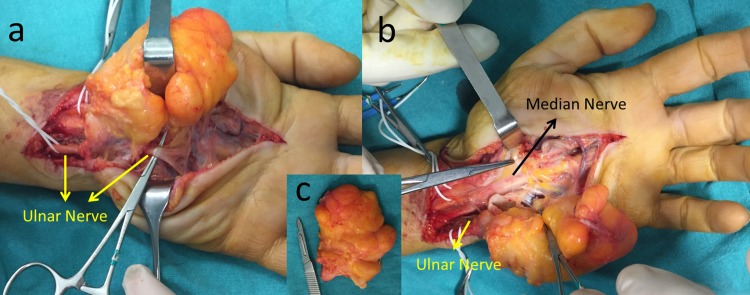
Intra-operative appearance Intra-operative appearance: (a) Compression of the ulnar nerve at Guyon's canal by the giant lipoma; (b) Compression of the median nerve at the carpal tunnel by the giant lipoma; (c) Appearance of the totally excised lipoma (6 x 5 x 4 cm).

Under ultrasound-guided brachial plexus block and tourniquet control, an incision extending to both Guyon’s canal and the carpal tunnel, through the palmar crisis was used. Compression of both the ulnar nerve in the Guyon’s canal and the median nerve in the carpal tunnel was observed intraoperatively (Figure 4).

**Figure 5:**
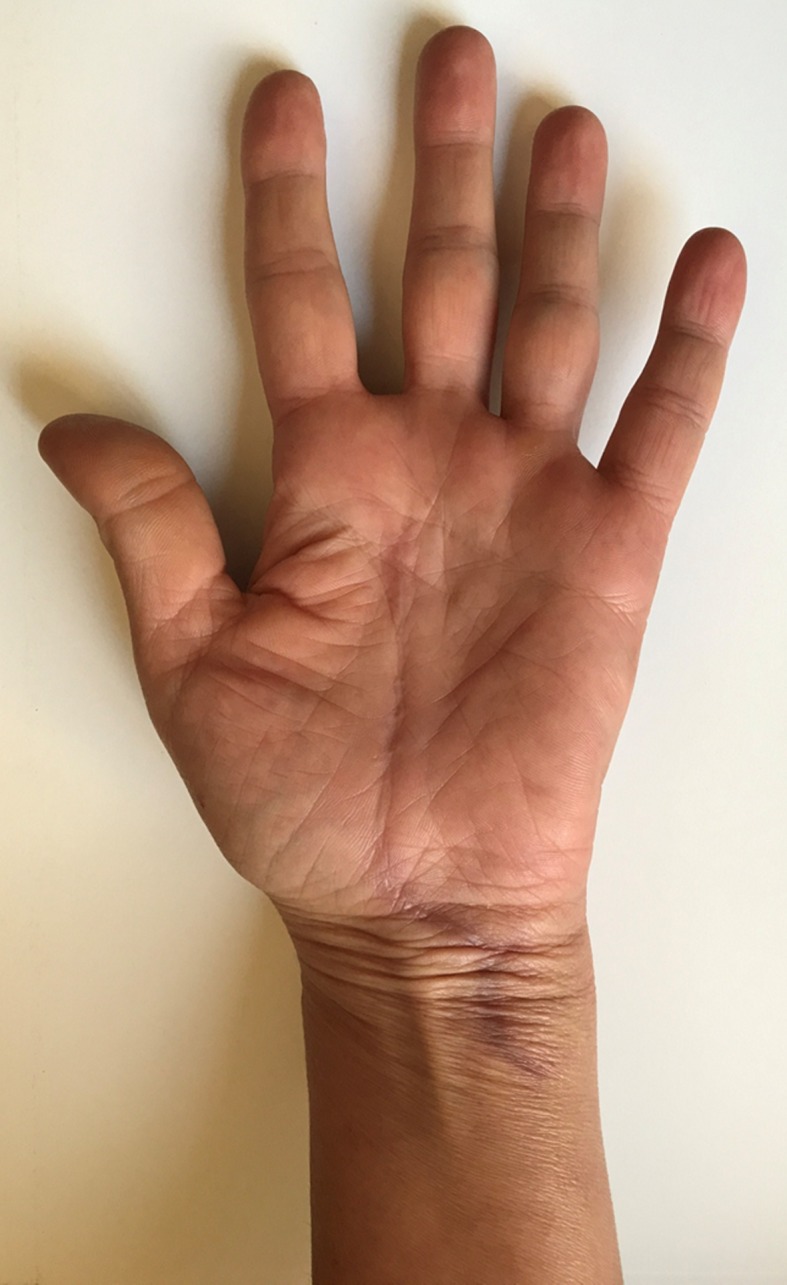
Clinical appearance of the patient’s hand at the final follow-up The clinical appearance of the patient’s hand at the final follow-up (nine months) was normal, with a well-healed incision.

The mass was totally excised. A histopathological examination confirmed the diagnosis of lipoma. The postoperative period was uneventful and sutures were removed on the 15th day after operation, and active hand and wrist movements were begun. The patient’s symptoms improved gradually and completely recovered at the second-month follow-up. At the final follow-up nine months postoperatively, the patient was free of pain and numbness. A control electrophysiological examination revealed normal findings. The disabilities of the arm, shoulder, and hand (Quik DASH) score was 2.27, with a hand grip strength of 23 kg on the affected side (left) and 28 kg on the contra-lateral side (right) (Figure 5).

## Discussion

The diagnosis of nerve compression syndromes of the upper extremity can usually be made based on typical history and physical examination findings. Additional electrodiagnostic studies may be used to confirm the clinical diagnosis and stage the degree of compression to decide the management strategy. Often, neither direct radiography nor other imaging modalities are ordered to rule out the possible presence of a space-occupying lesion in routine practice. While a subcutaneous lipoma could be identified easily during the physical examination, small-sized and deep-seated lipomas may be hidden and neglected easily. Furthermore, a lipoma may masquerade as thenar or hypothenar atrophy, which is an important physical examination finding in nerve compression syndromes.

Because the lipoma itself is painless, only compression neuropathy symptoms may be present, which is quite similar to a classical median or ulnar nerve compression syndrome. Consequently, the diagnosis may be missed or patients may receive inadequate treatment. Pogonis et al. described a case of carpal tunnel syndrome (CTS) that underwent repeated surgical release of the median nerve due to a missed lipoma [7]. Similarly, De Semet et al. reported another median nerve compression; that lipoma was discovered during the revision surgery in the carpal tunnel syndrome [8].

Conventional or idiopathic carpal tunnel syndrome is usually bilateral. Bagatur and Zorer reported 73% bilateral involvement in their series [9]. However, in case of a space-occupying lesion, the contralateral hand is almost always intact. Bagatur and Yalcinkaya reported two cases of CTS due to occult lipoma, and emphasized the physical examination of the asymptomatic hand and stressed on the importance of imaging studies in patients with unilateral symptoms that are usually not used in CTS [4].

In case of suspicion of a space-occupying lesion as the reason for nerve compression symptoms, imaging studies should be performed for the identification of the lesion. In direct radiography, small-sized lipomas can often be missed, while large lipomas may cause a radiolucent silhouette within the soft tissues. Magnetic resonance imaging (MRI) is the best examination method both for the differential diagnosis of the mass and for surgical planning. It is quite helpful to define mass characteristics and relationships with other anatomical structures [10]. An electrophysiological study is usually nonspecific; increased latency for both motor and sensory nerve conduction supports nerve entrapment neuropathy.

The treatment of a palmar lipoma is the marginal excision of the mass, without leaving a residue, and decompression of the involved nerves. For a complete removal of the mass, extended incisions may be required. Extended carpal tunnel incision is usually sufficient for the excision of palmar lipomas. In addition, a modified Brunner’s incision has been utilized in the literature [7]. The surgical incision should be as large as possible, which allows both the complete removal of the tumor and decompression of the affected nerve.

## Conclusions

In conclusion, a giant palmar lipoma that causes both median and ulnar nerve compression neuropathy is exceedingly rare in the literature. Particularly in patients with unilateral compression neuropathy symptoms, a physical examination should be performed thoroughly to seek space-occupying lesions of the hand. Further imaging examinations should be ordered.
